# Physiological and histopathological effects of electroporation pulse on stomach of rats

**DOI:** 10.1186/s12876-021-01924-0

**Published:** 2021-09-23

**Authors:** Yuchi Zhang, Xuan Han, Zhuoqun Li, Yu Zhang, Lihong Liang, Xiaoying Ma, Haonan Liu, Yihui Gao, Qingshan Li, Xue Chen, Yi Lv, Fenggang Ren

**Affiliations:** 1 National Local Joint Engineering Research Center for Precision Surgery and Regenerative Medicine, First Affiliated Hospital of Xi’an Jiaotong University, No. 277, West Yanta Road, Yanta District, Xi’an, 710061 China; 2 Department of Hepatobiliary Surgery, First Affiliated Hospital of Xi’an Jiaotong University, No.277, West Yanta Road, Xi’an, 710061 China; 3 Electrical Science and Technology Research Institute, School of Electrical Engineering, Xi’an Jiaotong University, No.28, West Xianning Road, Xi’an, 710049 China

**Keywords:** Electroporation pulse, Irreversible electroporation, Gastric tissue, Digestive function

## Abstract

**Background:**

Irreversible electroporation (IRE) is an emerging tissue ablation technique with widespread potential, especially for cancer treatment. Although the safety and efficacy of IRE for gastric tissue ablation have been demonstrated, there is a gap of knowledge regarding the effect of electroporation pulse (EP) on the physiology and histopathology of the stomach. This study applied EP to the stomach of healthy rats and investigated the digestive function, serum marker levels, and gastric tissue structure of EP-treated rats.

**Methods:**

Ninety male rats were divided into nine groups and examined up to 28 days post-treatment. A single burst of electroporation pulse (500 V, 99 pluses, 1 Hz, 100 µs) was delivered to the stomachs of rats using a tweezer-style round electrode. Gastric emptying, small intestinal transit, and gastric secretion were measured to evaluate the digestive function. Serum marker levels were determined using ELISA. Haematoxylin–eosin, Masson trichrome, and immunofluorescence were performed for histopathological analysis.

**Results:**

No  significant effect on gastric emptying or secretion was found post-EP, whereas the small intestinal transit decreased at 4 h and rapidly recovered to normal on 1-day post-EP. Further, serum TNF-α and IL-1β levels temporarily changed during the acute phase but returned to baseline within 28 days. Moreover, histopathological analysis revealed that cell death occurred immediately post-EP in the ablation area, whereas the gastric wall scaffold in the ablation region remained intact post-EP.

**Conclusions:**

This study demonstrates the safety and efficacy of EP on the physiology and histopathology of the stomach and lays a foundation for more comprehensive applications of this technique.

## Background

Irreversible electroporation (IRE) is a tissue ablation technique that allows delivering a burst of microsecond electric field pulses with specific characteristics, which yields the disruptions of cell membrane [1]. Usually, IRE deploys a square wave with a pulse width on the order of tens to hundreds of microseconds, while the electric field intensity in the ablation area is approximately 700–1000 V/cm, which was also called electroporation pulse (EP) [2]. The cell transmembrane potential changes under the effect of EP, destroying the stability of the cell membrane and local environmental homeostasis, ultimately leading to cell death. Therefore, EP has been recognised as an emerging tissue ablation technique and applied to treat pancreas, prostate, and liver cancer in clinical practice [3, 4].

The duration of a single EP is ultra-short; hence, the heat generated during the IRE process quickly diffuses or is absorbed. Therefore, the cumulative temperature of the tissue is negligible when IRE is achieved, while no thermal damage to the tissue is caused [5]. EP can inactivate malignant cells in situ rapidly while sparing tissue scaffolds such as the extracellular matrix, vascular wall, and nerve fibre. Furthermore, EP is not affected by the heat sink effect, so the blood vessel adjacent to the ablation area will be unaffected [6]. These advantages of IRE prominently expanded the application of tissue ablation techniques, especially for heat-sensitive structures, which are usually out-of-reach for tissue ablation.

Typically, heat energy is not easy to control for thermal-basis tissue ablation, potentially leading to serious side effects when applied to heat-sensitive structures [7]. On the one hand, perforation or irreversible thermal damage may occur with a high intensity of heat energy. However, a lower intensity may lead to residual tumour formation and decrease treatment efficacy [8]. Based on the non-thermal features of EP, several studies reported EP as a potential ablation method for cavity organs such as the digestive tract. The safety and efficacy of IRE for the bile duct [9, 10], heart [11], and urinary tract [12] have been demonstrated in animal studies. Lee et al. have investigated the characterisation of IRE on the stomach in a rat model and demonstrated that IRE could potentially be used as a minimally invasive treatment for early gastric cancer. In our previous studies, we have designed endoscopic catheter-based ablation electrodes and investigated the safety and efficacy of endoscopic IRE for gastric tissue ablation in animal models [8, 13]. However, in the stomach, due to the unique anatomy and function of this digestive organ, there is a lack of basic research on the biological effects and outcomes of EP on the physiology and histopathology of the healthy stomach. Therefore, this study aimed to investigate the biological effects and outcomes of EP in terms of digestive function, serum marker levels, and gastric tissue structure in a rat model.

## Methods

### Animal care and ethics

Ninety male Sprague Dawley rats (180–280 g) were purchased and kept at the Experimental Animal Center of Xi’an Jiaotong University. The animals were maintained in standard day and night cycle (12 h light to 12 h dark) and environment temperature (25 ± 2 °C) with free access to food and water. All the animals were kept in the same condition. The study protocols were approved by the Institutional Animal Care and Use Committee of Xi’an Jiaotong University (No. XJTU2018-463).

### Experimental protocol

The animals were randomly divided into eight groups according to their sacrifice time: 0 h, 4 h, 1 day, 3 days, 7 days, 14 days, and 28 days post-EP and control groups. Random numbers were generated using a computer-based random order generator. The 0 h group contained 6 rats for histopathological analysis, while the other groups contained 12 rats for physiological (n = 6) and histopathological analysis (n = 6). No treatment was performed on the control group, which was used for collecting physiological and histopathological reference data.

**Fig. 1:**
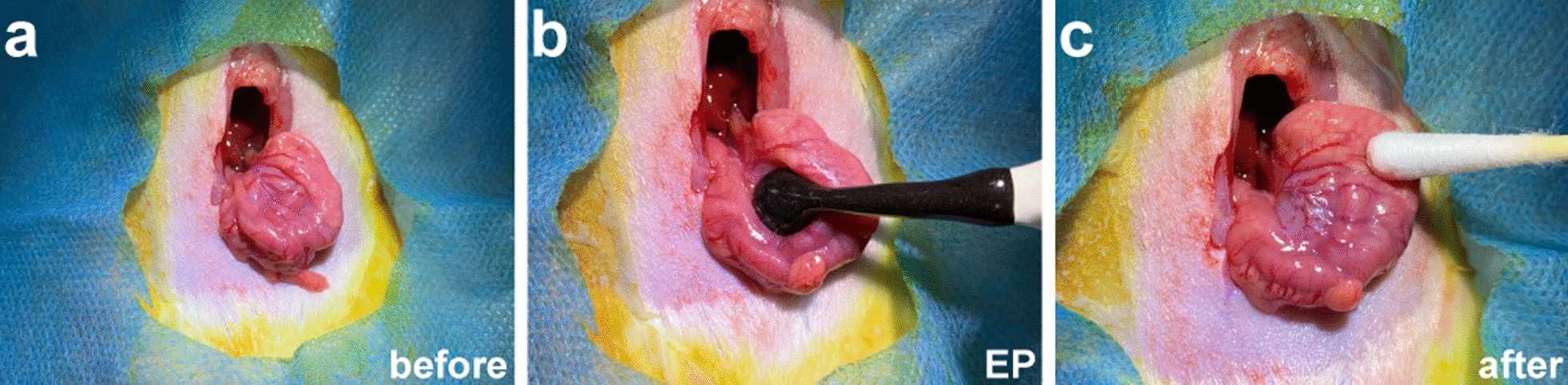
The experimental procedure of EP on the stomach of rats. **a** The serosal face of posterior wall of stomach before EP; **b** A Tweezer-style electrode was used to clamp the full thickness from the anterior to the posterior wall in the middle of the gland stomach near the cardia during EP; **c** The serosal face of anterior wall of the stomach after EP

All animals fasted for 24 h before the operation. Inhalational anaesthesia was maintained with isoflurane (RWD, Shenzhen, China). The oxygen and isoflurane flow rates were set to 0.6 L/min and 2 L/min, respectively. The stomach was exposed with a 2–3 cm incision along the midline under the xiphoid. A Tweezer-style round electrode (BTX Platinum Tweezertrode, 5 mm in Diameter, Model: 45-0489, Harvard Bioscience, Holliston, MA, USA) was used to clamp the full thickness from the anterior to the posterior wall in the middle of the gland stomach near the cardia, maintaining the gastric wall structurally intact without punctures. The spacing between the pair of electrodes was about 2–3 mm. The schema for the operation procedure is shown in Fig. 1.

After that, a single burst of EP was delivered to the stomach of rats in the experimental groups using a commercial electroporation system (BTX ECM830 square wave, Harvard Bioscience, Holliston, MA, USA). The EP parameters were set to 500 V pulse voltage, 100 µs pulse width, 1 Hz pulse frequency, and 99 pulse number. These parameters were set based on numerical analysis for electrical and thermal damage from our previous work [5], which reportedly causes significant electrical damage on the gastric wall without thermal damage. After the operation, the behaviour and feeding of animals were observed daily.

### Digestive function measurement

Gastric emptying and small intestinal transit were measured by intragastric administration of the phenol red–methylcellulose (PR-HPMC) method, as previously reported [14]. PR-HPMC food was prepared by dissolving 50 mg of phenolic red (A100882, Sangon Biotech, Shanghai, China) in 100 mL of 1.5% hydroxypropyl methylcellulose water solution (A600529, Sangon Biotech, Shanghai, China). The stomach/small intestine specimens and gastric contents were harvested at 0.5 h after gavage. The supernatant of the gastric content was analysed with a microplate reader (Varioskan Flash, Thermo Fisher Scientific, Waltham, MA, USA) at 560 nm to determine the OD values. Then, the gastric emptying rate was calculated as percent emptying, which was calculated as follows: 1 − absorbance of the test sample/absorbance of the standard. The OD values of gastric contents from rats euthanised immediately after PR-HPMC gavage served as a standard. The furthest point of phenol red migration in the small intestine was verified, and the distance between this point and the initial intestinal segment was recorded as *D*. The overall length of small intestine specimens was determined as *L*. Thus, the small intestine transit was calculated as *D/L*. Gastric secretion was evaluated using pyloric ligation. The stomach was ligated between the pylorus and duodenum, followed by fasting for 4 h. Then, the gastric specimens and contents were collected, and the volume of the gastric contents was measured as gastric juice (mL).

### Serum marker determination

Blood samples were collected from inferior vena cava by open surgery under inhalational anaesthesia at each indicated time point, as described before, after which the rats were sacrificed due to massive blood loss, and tissue samples were collected. The serum levels of prostacyclin I2 (PG I_2_) (Rat Prostaglandin E2 ELISA Kit, Cusabio, Wuhan, China), prostaglandin E2 (PG E_2_) (Rat Prostacycline ELISA Kit, Cusabio, Wuhan, China), nitric oxide (NO) (Nitric Oxide Kit, Jiancheng Bioengineering, Nanjing, China), ghrelin (ELISA Kit For Ghrelin, USCN Kit, Wuhan, China), tumour necrosis factor-alpha (TNF-α) (Rat TNF-α ELISA Kit, Thermo Fisher Scientific), interleukin 1β (IL-1β) (Rat IL-1β ELISA Kit, Multi Sciences Biotech, Hangzhou, China), interleukin-6 (IL-6) (Rat IL-6 ELISA Kit, Multi Sciences Biotech, Hangzhou, China), and interleukin-10 (IL-10) (Rat IL-10 ELISA Kit, Multi Sciences Biotech, Hangzhou, China) were determined using commercially available ELISA kits following the manufacturer’s instructions.

### Histopathological analysis

Stomach specimens were dissected along the greater curvature. After washing with phosphate buffer saline, specimens were fixed in paraformaldehyde, embedded in paraffin, and sliced into 4 μm sections. Each sample was stained with haematoxylin–eosin (H&E) (G1005, Servicebio, Wuhan, China) and Masson trichrome (G1006, Servicebio, Wuhan, China) according to the manufacturer s instructions. Moreover, terminal deoxynucleotidyl transferase-mediated nick end labelling (TUNEL) (In Situ Cell Death Detection Kit, Roche, Basel, Switzerland) and the expression of E-cadherin (1:200) (GB12082, Servicebio, Wuhan, China), β-Catenin (1:200) (G12015, Servicebio, Wuhan, China), CD117 (1:2000) (GB11073-2, Servicebio, Wuhan, China), PGP9.5 (1:100) (GB11159-1, Servicebio, Wuhan, China), and proliferating cell nuclear antigen (PCNA) (1:100) (GB11010, Servicebio, Wuhan, China) were detected by immunofluorescence following the manufacturer’s instructions. Histopathological analysis was performed by two experienced pathologists unaware of the group allocation.

### Statistical analysis

All statistical analyses were performed using GraphPad Prism 8.0 for Windows (GraphPad Software Inc., La Jolla, CA, USA). Quantitative variables were expressed as means ± SD and analysed by Student’s t-test or one-way ANOVA. All statistical tests were bilateral, and the results were considered statistically significant at *P* < 0.05.

## Results

### Digestive function

The changes in digestive function post-EP are summarised in Table 1. Compared with the control group, there was no significant difference among the groups in gastric emptying levels. The small intestine transit decreased from 67.71 ± 6.18% to 33.25 ± 10.49% (*P* < 0.001) 4 h post-EP, but it rapidly recovered to 55.67 ± 13.58% at 1 days post-EP. There was no significant difference in small intestine transit among groups from 1 to 28 days post-EP, while the data fluctuates during these times. As for gastric secretion, the volume slightly decreased after EP; however, the difference was not statistically significant.

**Table 1:** Changes in digestive function post-electroporation pulse

Post-electroporation pulse time	Gastric emptying (%)	Small intestine transit (%)	Gastric acid secretion (mL)
Control	79.82 ± 8.26	67.71 ± 6.18	5.29 ± 2.06
4 h	65.43 ± 13.59	33.25 ± 10.49***	3.12 ± 1.70
1 day	60.62 ± 15.54	55.67 ± 13.58	2.72 ± 1.32
3 days	69.26 ± 9.99	59.12 ± 4.84	4.38 ± 1.34
7 days	66.75 ± 10.65	63.17 ± 10.93	3.00 ± 2.89
14 days	71.65 ± 6.32	54.85 ± 8.98	3.30 ± 0.90
28 days	59.13 ± 23.52	52.73 ± 14.53	2.96 ± 1.67

***P < 0.001. Compared with control group

### Serum marker analysis

The changes observed in several serum markers after EP are summarised in Table 2. Serum TNF-α and IL-1β were higher in the post-EP group than in the control, but the difference was not statistically significant. IL-10 levels decreased from 22.30 ± 12.08 to 8.47 ± 6.70 pg/mL (*P* < 0.05) 4 h post-EP, further decreased to 4.71 ± 8.97 pg/mL (*P* < 0.01) on day 1 post-EP (*P* < 0.001), and recovered to 21.57 ± 15.02 pg/mL 3 days post-EP. The serum IL-6 level reached a peak (131.7 ± 104.2 pg/mL vs. 10.28 ± 29.23 pg/mL, *P* < 0.001) 3 days post-EP, while no statistically significant difference was observed among other groups. Serum PGI_2_ and PGE_2_ levels increased and reached a peak at 14 days (*P* < 0.001). The serum ghrelin level decreased within 7 days post-EP, reaching statistical significance (10,136 ± 2484 pg/mL vs. 5268 ± 1781 pg/mL, *P* < 0.001) 7 days post-EP and gradually recovered to baseline within 14 days. NO significantly increased to 1.543 ± 0.2082 µmol/L 3 days post-EP, but returned to normal within 14 days post-EP.

**Table 2:** Changes in serum marker levels post-electroporation pulse

Post-electroporation pulse time	TNF-α (pg/mL)	IL-1β (pg/mL)	IL-10 (pg/mL)	IL-6 (pg/mL)	PG I_2_ (ng/mL)	PG E_2_ (pg/mL)	NO (µmol/L)	Ghrelin (pg/mL)
Control	0.35 ± 0.87	35.98 ± 48.16	22.30 ± 12.08	10.28 ± 29.23	0.51 ± 0.15	1.31 ± 0.46	1.01 ± 0.17	10,136 ± 2484
4 h	2.10 ± 2.50	94.17 ± 85.63	8.47 ± 6.70*	3.25 ± 5.18	0.58 ± 0.21	1.65 ± 0.55	0.99 ± 0.15	11,590 ± 2828
1 day	1.92 ± 2.73	91.91 ± 77.72	4.71 ± 8.94**	19.44 ± 37.99	0.62 ± 0.10	1.45 ± 0.38	0.98 ± 0.21	9184 ± 3001
3 days	1.11 ± 2.06	63.81 ± 83.63	21.57 ± 15.02	131.70 ± 104.20***	0.76 ± 0.50	1.49 ± 0.67	1.54 ± 0.21**	7534 ± 965
7 days	3.10 ± 4.05	76.68 ± 87.24	23.68 ± 12.01	11.23 ± 29.34	0.85 ± 0.50	1.72 ± 0.94	1.34 ± 0.26*	5268 ± 1781**
14 days	3.02 ± 3.70	112.00 ± 114.60	13.88 ± 10.71	4.50 ± 9.64	1.44 ± 1.28***	2.97 ± 2.04***	1.00 ± 0.55	7589 ± 3155
28 days	3.38 ± 3.31	38.39 ± 57.63	12.55 ± 10.98	8.04 ± 16.64	0.37 ± 0.11	1.01 ± 0.47	0.95 ± 0.28	7989 ± 3185

*P < 0.05; **P < 0.01; ***P < 0.001. Compared with control group

### Gross pathology

**Fig. 2:**
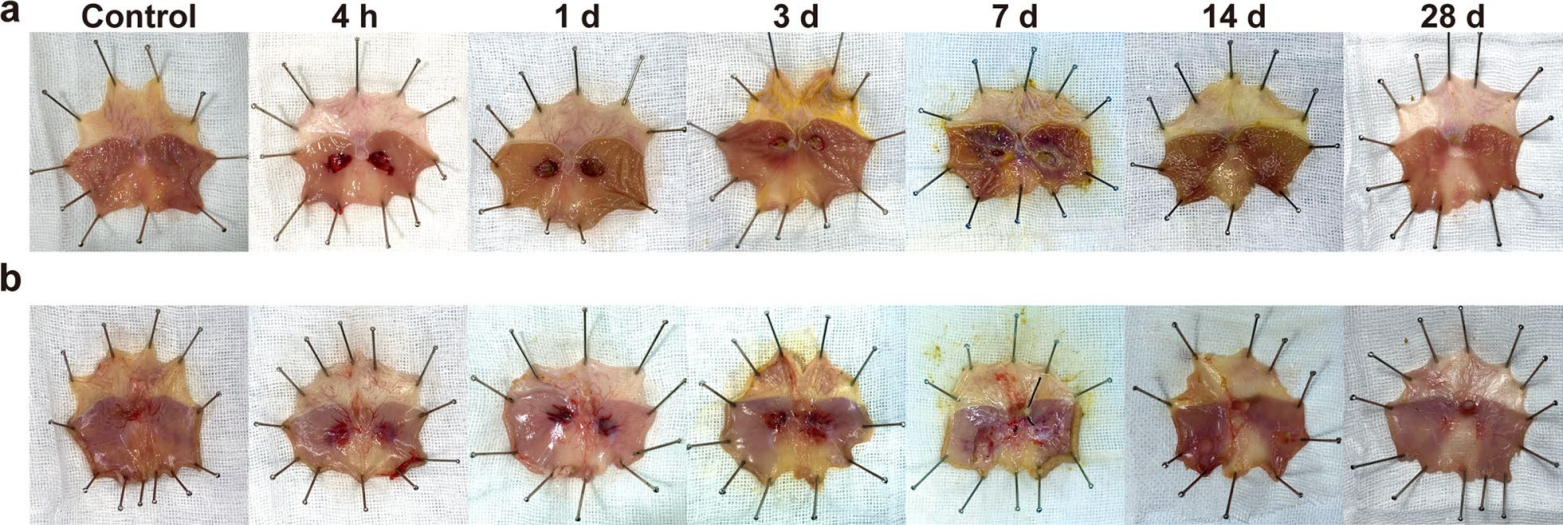
Gross appearance of the rat gastric wall post-treatment with electroporation. **a** mucosal surface; **b** serosal surface

Images of the gross pathology are shown in Fig. 2. Clearly demarcated lesions with congestion were observed on the treated mucosa immediately post-EP (Fig. 2a). The shape and size of the lesions were broadly consistent with the electrode. On day 1 post-EP, the mucosa had sloughed off, and the lesions turned into ulcerations. The lesions started to recover from day 3 to day 7 post-EP. On day 28 post-EP, the mucosal face of the lesions was recovered and showed a statistically not significant difference compared to the surrounding normal tissue. On the serosal face of the stomach (Fig. 2b), the ablation region turned darker after EP without bleeding or perforation. The serosal lesions became smaller, lighter in colour, and completely healed within 28 days post-EP.

### Histopathology

**Fig. 3:**
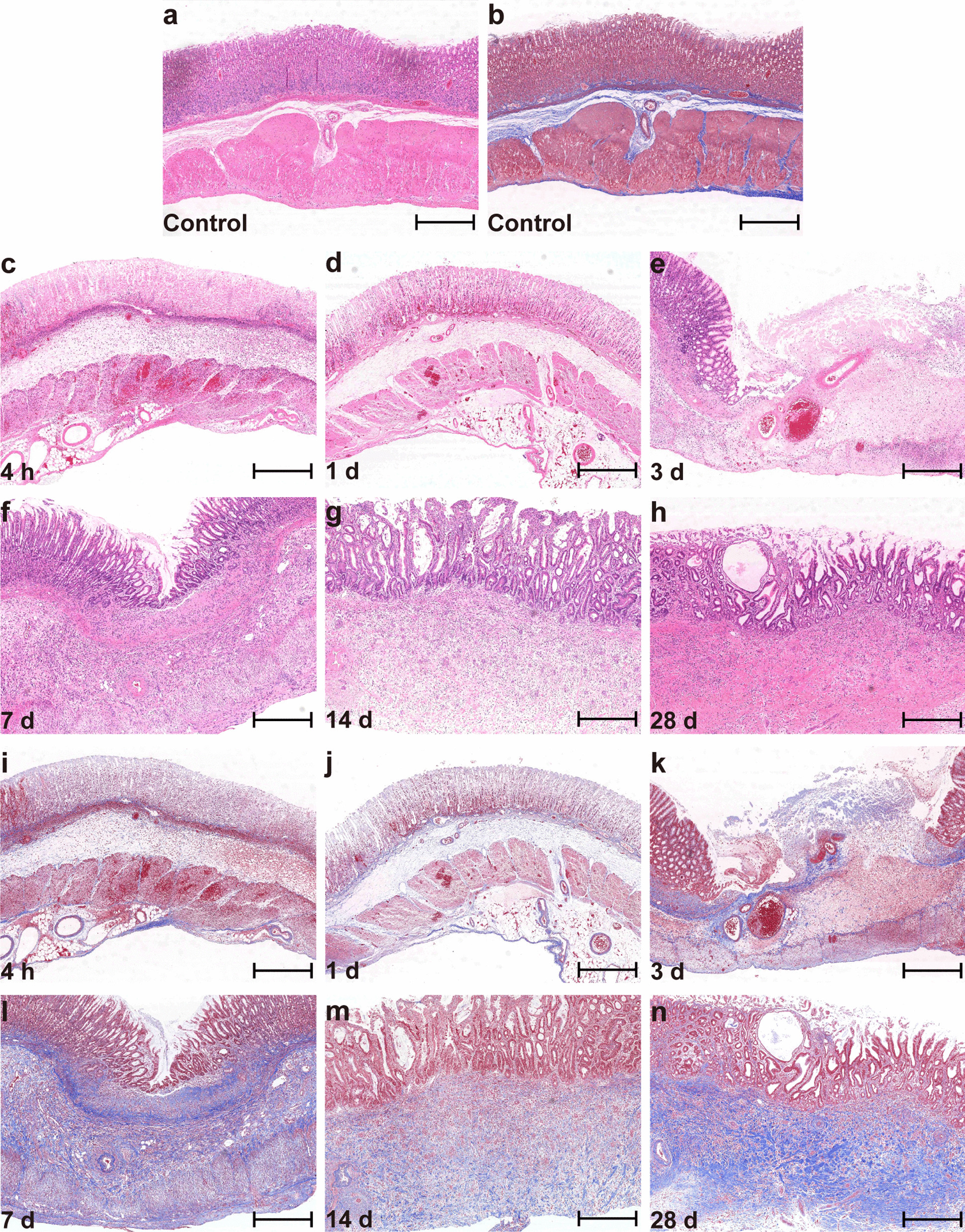
Histopathology of the rat gastric wall post-treatment with electroporation. **a** Normal; **b** Normal; **c–h** H&E staining from 4 h to 28 days post-EP; **i**–**n** Masson trichrome staining from 4 h to 28 days post-EP. Scale bar: 500 μm

Figure 3 shows the H&E staining of the gastric wall 28 days post-EP. A well-circumscribed ablation area was immediately produced within 4 h post-EP. The glandular epithelial structure disappeared with massive inflammatory cell infiltration in the mucosal layer, while the muscular propria remained intact with local hyperaemia or haemorrhage (Fig. 3c). Necrosis on the entire gastric wall was observed at 1 day post-EP. The glandular epithelial cells and smooth muscle cells disappeared, accompanied by substantial inflammatory cell infiltration, forming an inflammatory response zone (Fig. 3d). The necrotic mucosa then fell off, resulting in ulceration on the gastric wall 3 days post-EP (Fig. 3e). Meanwhile, cells around the ablated area proliferated and gradually grew into the necrotic area starting on day 3 post-EP, and a complete repair of the gastric wall occurred within 14–28 days post-EP (Fig. 3f–h).

Masson’s trichrome staining indicated that the gastric wall scaffold in the lesion remained intact after EP (Fig. 3i–n). In the 3-days sample, Masson trichrome staining showed that the muscularis mucosa, submucosa, and muscularis propria were replaced by collagen, suggesting that the muscular layer repair is mainly caused by fibrosis. Meanwhile, no significant change was observed in the gastric wall thickness within 14–28 days post-EP.

**Fig. 4:**
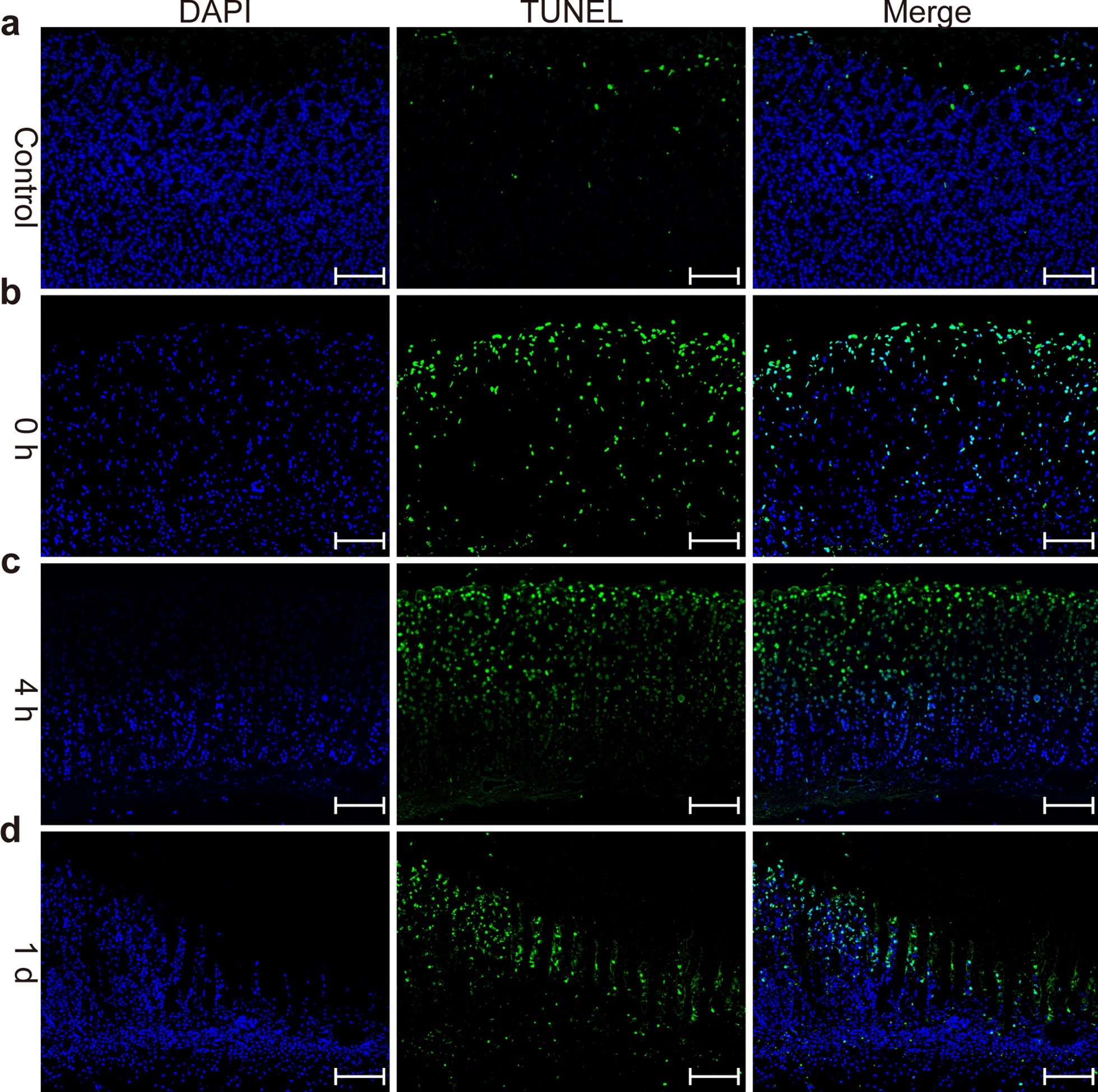
Histopathology analysis of TUNEL in gastric mucosa post-electroporation (EP), as determined by immunofluorescence (from left to right: DAPI, TUNEL, and Merge). **a** Control; **b** 0 h post-EP; **c** 4 h post-EP; **d** 1 days post-EP. Scale bar: 100 μm

For TUNEL, a massive number of positive cells were observed in the mucosa and muscularis propria immediately post-EP (Fig. 4b). Viable epithelium decreased 4 h post-EP, and the transition of TUNEL-positive cells from the mucous epithelium to the muscularis mucosa increased (Fig. 4c). Complete ablation of the mucosa and muscularis propria was observed within 12–24 h (Fig. 4d).

**Fig. 5:**
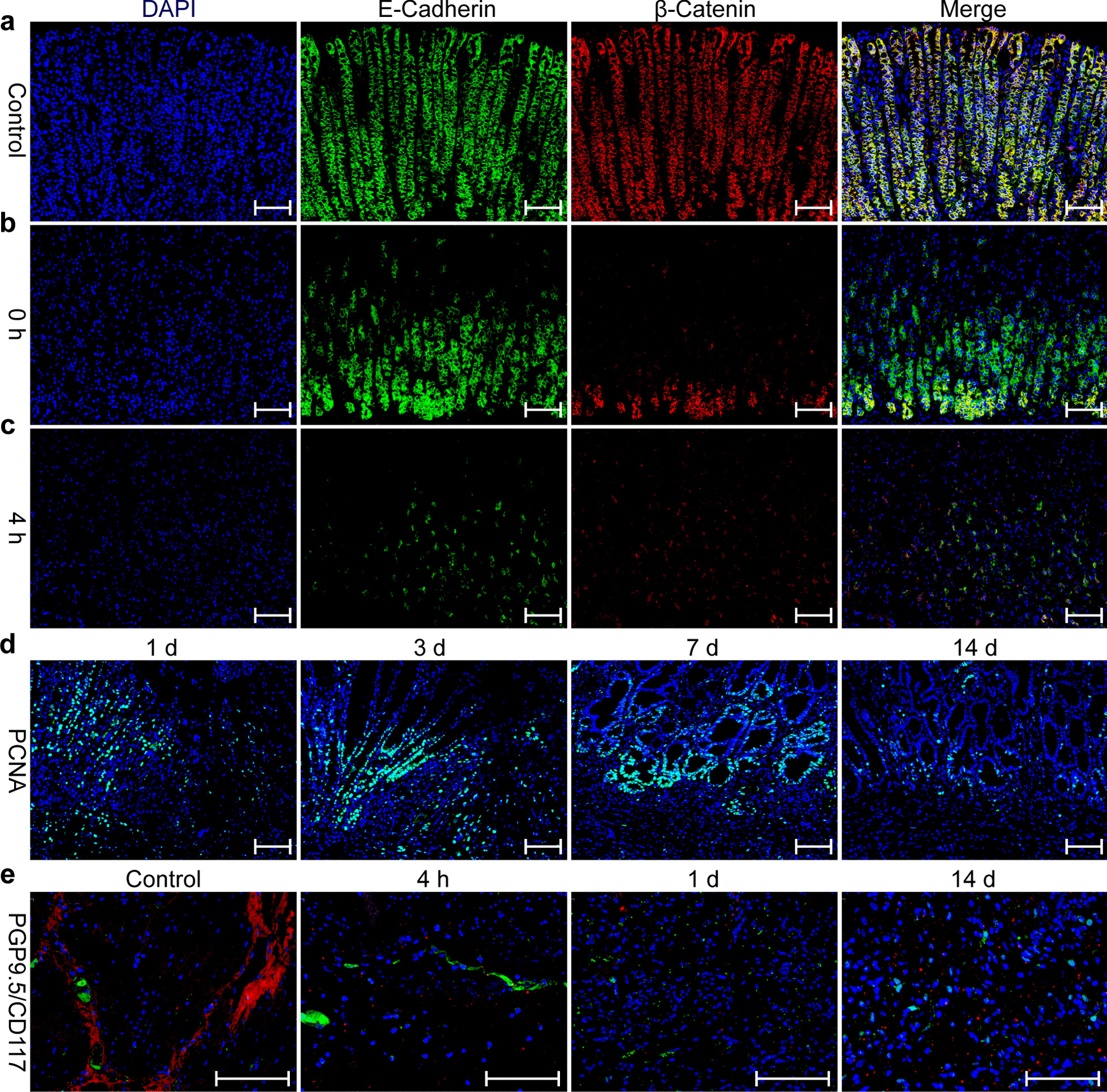
Histopathology analysis of E-cadherin (green), β-catenin (red) (**a**–**c**), and PCNA (green) (**d**) in the gastric mucosa, and PGP9.5 (green), CD117 (red) (**e**) in gastric serosa following electroporation pulse, as determined by immunofluorescence. Scale bar: 100 μm

The expression of E-cadherin and β-catenin in the ablated area was lower than that in normal tissue immediately and 4 h post-EP, but rarely occurred in the 24 h samples, indicating that the epithelium intercellular junction was severely damaged by EP (Fig. 5a–c).

At the edge of the ablation area, PCNA staining showed an increase in the number of positive cells in the gastric fundus gland, suggesting that mucosal cells began the proliferation and repair process 3 days post-EP (Fig. 5d). By day 7, PCNA showed increased expression in the fundus gland, suggesting active stem cell proliferation. Along with migration of the immature epithelium, mucosal regeneration and repair were completed by day 14 post-EP. Moreover, EP successfully induced a reduction of CD117 and PGP9.5 positive cells by day 1 in the muscularis propria, indicating the injury of Cajal cells and neurons by EP. At 14 days post-EP, no CD117- and PGP9.5-positive cells were observed in the regenerated muscularis propria (Fig. 5e).

## Discussion

EP-based treatment is an attractive candidate for tumour ablation, especially for those located in heat-sensitive areas, such as blood vessels, nerves, the bile duct, and the gastrointestinal tract. Although the safety and efficacy of EP for gastric tissue ablation have been demonstrated using endoscopy in our previous work, the basic research on the physiological and histopathological changes of the stomach post-EP is limited. In this study, EP was performed on the stomach of healthy rats to evaluate the effect of EP on the digestive function, serum marker levels, and gastric structure over 28 days. The safety and efficacy of EP in terms of both physiology and histopathology have been confirmed in a rat model. The digestive function and serum markers changed temporarily in the acute phase but soon returned to normal within 28 days. The gastric wall remained intact without bleeding or perforation after EP. This study confirmed the feasibility and safety of EP-based therapy for the stomach in a rat model, which provides evidence for future use in clinical practice.

The stomach is a vital organ for digestion and secretion. Gastric emptying, small intestinal transit, and gastric secretion are essential indicators of digestive function [15, 16]. After EP, no significant change was observed in terms of gastric emptying or secretion. The small intestine transit decreased 4 h post-EP, whereas it recovered to normal 1 day later and remained normal throughout the 28 days of observation. This study confirmed that EP has a limited impact on digestive functions in a rat model; thus, EP is a safe ablation method regarding digestive function.

Serum markers are reportedly dynamic in rat models. Post-EP, inflammation factors may be regulated by multiple factors. By and large, anti-inflammatory factors (IL-10) tended to be reduced within 1 days and recovered later, while proinflammatory factors (TNF-α, IL-1β, IL-6) showed an opposite trend, consistent with the gastric mucosa gastric mucosa by inhibiting acid secretion, promoting mucus generation, and increasing mucosal blood flow [17]. The prostaglandin level in the treatment group tended to rise and reached a peak 14 days post-EP. In addition, serum NO level, which can improve microcirculation and mucosal reconstruction, reached a peak on day 3, and then returned to normal on day 14, consistent with the histopathology of mucosal regeneration. Ghrelin increased from day 7 to day 14 post-EP, during which the mucosa regenerated rapidly.

The thermal-basis ablation technique could generate coagulation necrosis instantly after ablation, including in a peripheral transition zone around the ablation region due to the temperature gradient [18]. All these changes can be determined by gross observation. However, a non-thermal ablated lesion was caused without evident coagulation necrosis on gross inspection [19, 20]. The cell viability, gross pathology, and histopathology show dynamic changes post-EP, which vary in different tissues [21]. This study comprehensively analysed the gross changes of the mucosal and serosal surface of the stomach post-EP. The ablated mucosa fell off within 3 days post-EP; thus, an artificial ulcer was formed on the gastric wall. Afterward, the regeneration process began, and the gastric wall was repaired within 14–28 days.

Ensuring the integrity of the gastric wall is vital for evaluating the safety of EP. Phillips et al. studied the influence of IRE on the small intestine structure of rats and demonstrated that the small intestine could be ablated completely by IRE without noticeable gastrointestinal side effects [22]. The epithelium starts repairing 3 days after surgery. In this study, EP was applied to the stomach, and the change in gastric structure within 24 h was evaluated. The demarcated lesions with congestion on both gastric mucosa and serosa were caused by EP, which may be related to the vascular lock-in effect of EP [6]. Histopathology showed immediate death of cells contacting the electrode after EP and complete ablation of the mucous layer at 24 h. The 0 h and 4 h samples also revealed massive numbers of positive cells in the TUNEL assay, indicating that cell apoptosis started as early as 0 h and reached a peak at 24 h post-EP in the ablated area. The E-cadherin and β-catenin complex are vital for the tight junction between epithelial cells, which is crucial for the formation of the gastric mucosal barrier that protects the mucosa from gastric acid [23, 24]. This study revealed that the expression of E-cadherin and β-catenin decreased in mucosal epithelial cells immediately post-EP, suggesting a tight junction break and destruction of the mucosal barrier, ultimately promoting mucosal damage due to gastric acids.

There were several limitations to this study. First, normal gastric tissue differs from tumour tissue; hence, the efficacy of EP for tumour ablation has not been sufficiently elucidated. Second, since the digestive function is closely related to nerve distribution, future studies are needed, including additional treatment locations. Third, given the disparity between humans and rats, the practical treatment process cannot be thoroughly evaluated in this study and requires further investigation in large animal models and/or humans.

## Conclusions

This study demonstrated the safety and efficacy of EP on the physiology and histopathology of rat stomachs. The digestive function was slightly affected but soon returned to normal. The gastric wall remains intact, and the mucosa can be ablated using EP without perforation or bleeding. This study confirmed that EP is an attractive candidate for gastric tissue ablation and has laid the foundation for the broader use of this technique in the future.

## Data Availability

The datasets used and/or analysed during the current study are available from the corresponding author on reasonable request.

## Abbreviations

IRE: Irreversible electroporation
EP: Electroporation pulse
TNF-α: Tumour necrosis factor-alpha
IL-1β: Interleukin 1β
IL-10: Interleukin-10
IL-6: Interleukin-6
PG I_2_: Prostacyclin I_2_
PG E2: Prostaglandin E2
NO: Nitric oxide
